# Exploring the shared decision making process of caesarean sections at a teaching hospital in Ghana: a mixed methods study

**DOI:** 10.1186/s12884-023-05739-7

**Published:** 2023-06-08

**Authors:** Kwaku Asah-Opoku, Aisha N. Onisarotu, Mercy A. Nuamah, Elena Syurina, Kitty Bloemenkamp, Joyce L. Browne, Marcus J. Rijken

**Affiliations:** 1 Department of Obstetrics and Gynecology, University of Ghana Medical School, Accra, Ghana; 2 Korle-Bu Teaching Hospital, Accra, Ghana; 3 Department of Obstetrics, Division Woman and Baby, Wilhelmina’s Children Birth Centre, University Medical Centre Utrecht, Utrecht University, Utrecht, the Netherlands; 4 Athena Institute, Faculty of Earth and Life Sciences, Vrije University, Amsterdam, Netherlands; 5 Julius Global Health, Julius Centre for Health Sciences and Primary Care, University Medical Centre, Utrecht University, Utrecht, the Netherlands

**Keywords:** Caesarean section, Healthcare professionals, Mothers, Medical indication, Shared decision-making, Ghana

## Abstract

**Background:**

Caesarean section (CS) rates are rising. Shared decision making (SDM) is a component of patient-centered communication which requires adequate information and awareness. Women in Ghana have varying perceptions about the procedure. We sought to explore mothers’ knowledge. perceptions and SDM-influencing factors about CSs.

**Methods:**

A transdisciplinary mixed-methods study was conducted at the maternity unit of Korle-Bu Teaching Hospital in Accra, Ghana from March to May, 2019. Data collection was done in four phases: in-depth interviews (*n* = 38), pretesting questionnaires (*n* = 15), three focus group discussions (*n* = 18) and 180 interviewer administered questionnaires about SDM preferences. Factors associated with SDM were analyzed using Pearson’s Chi-square test and multiple logistic regression.

**Results:**

Mothers depicted a high level of knowledge regarding medical indications for their CS but had low level of awareness of SDM. The perception of a CS varied from dangerous, unnatural and taking away their strength to a life-saving procedure. The mothers had poor knowledge about pain relief in labour and at Caesarean section. Health care professionals attributed the willingness of mothers to be involved in SDM to their level of education. Husbands and religious leaders are key stakeholders in SDM. Insufficient consultation time was a challenge to SDM according to health care professionals and post-partum mothers. Women with parity ≥ 5 have a reduced desire to be more involved in shared decision making for Caesarean section. AOR = 0.09, CI (0.02–0.46).

**Conclusion:**

There is a high knowledge about the indications for CS but low level of awareness of and barriers to SDM. The fewer antenatal care visits mothers had, the more likely they were to desire more involvement in decision making. Aligned to respectful maternity care principles, greater involvement of pregnant women and their partners in decision making process could contribute to a positive pregnancy experience. Education, including religious leaders and decision- making tools could contribute to the process of SDM.

**Supplementary Information:**

The online version contains supplementary material available at 10.1186/s12884-023-05739-7.

## Background

Globally, the rate of caesarean sections (CSs) has rapidly increased over the years and exceeded the World Health Organization (WHO) recommended rate of 10 to 15% [1]. This rise has been universal; in low-, middle- and high-income countries. There is no health benefit to CSs that are carried out without indication except in instances like previous traumatic vaginal births with a sequela of psychological and psychiatric problems [1–3]. The increase in CS rates has also been observed in Ghanaian hospitals. For instance, at the Korle-Bu Teaching Hospital, a tertiary referral hospital in Accra, Ghana, the CS rate has increased towards 40 to 50% in the last decade [4] while the national Caesarean section birth rate is 16% [5].

Despite the high rates of CSs, knowledge on this procedure is quite low among women in Ghana [6]. A study in Kumasi found that majority of Ghanaian women were not aware of the indication for their CS [6]. Furthermore, heterogeneity in perceptions and beliefs about CS have been reported; some women thought that undergoing CS was dangerous to mother and child [6] or CSs were done because women were lazy. Others thought CSs were only done in order to save the lives of mothers and babies [7, 8]. These differing perspectives/opinions on CSs highlight a knowledge gap that needs to be addressed. It is clear that prior to performing CSs, women are not fully informed/properly counselled. Exploration of this knowledge gap will be helpful in formulating of public health education policies on this issue.

According to WHO’s guidelines on intrapartum care, one of the prerequisites for a positive childbirth experience is effective communication and involvement of a mother in the decision-making process [9–11]. Shared decision‐making involves health professionals and patients/clients working together to achieve true person‐centered health care [12]. Numerous advantages of shared decision making (SDM) have been reported, including increased patient involvement and confidence in clinical decisions, improved patient knowledge and more realistic expectations [11]. Yet, barriers to SDM exist, both on health system’s level if there is inadequate time, numbers of staff or enabling environment, as well as patient-related or cultural factors such as low health literacy level of women or cultures that do not value a person making an autonomous decision as important [11].

One way to address the increasing CS rates and improve respectful maternity care in Ghana is to involve women in the decision making concerning CSs [13, 14]. If mothers are more aware of the indications, risks and benefits of CS, they could make a more informed decision together with health care professionals. These factors may give directions for future interventions to reduce unnecessary CSs [15] and increase women’s satisfaction [14]. Therefore, the aim of this study is to explore the perspectives of mothers and health care professionals at a teaching hospital in Ghana about CS to understand the current SDM practices.

## Methods

### Study design

**Fig. 1:**
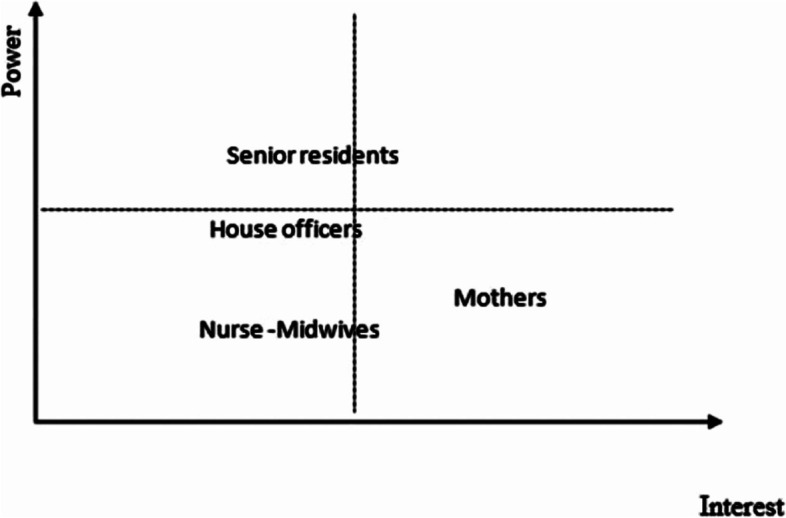
Analysis of stakeholders involved in the shared decision-making process

This was a transdisciplinary mixed-methods research carried out from 15^th^ March to 15^th^ May 2019 at Korle-Bu Teaching Hospital in Accra, Ghana. For transdisciplinary research, we ensured that the study was responsive to what stakeholders, including caregivers and mothers, found important so they could develop co-ownership of the project. This was achieved by researchers (epidemiologists, anthropologists and statisticians) collaborating with healthcare givers (nurse/midwives, house officers and residents) to ascertain the interest of mothers throughout the proposal development.as shown in Fig. 1 [16–18].

The SDM process is also based on the fact that the power (decision) of the healthcare professionals to carry out a cesarean section is made in agreement with (interest) the mother.

Following the co-design method, which aimed to improve healthcare through patient involvement, the results of each research phase fed into the next phase [18]. This study was reported according to the STROBE and COREQ guidelines for observational studies [19, 20].

### Setting

Korle-Bu Teaching Hospital is a tertiary hospital in Accra, Ghana and is one of the main referral centres in Greater Accra and neighbouring regions. Korle-Bu Teaching Hospital has an average of 10 000 births annually, with a CS rate rising from below 40% in 2011 to over 49% in 2022 [4, 21, 22]. Of all CS births, 42.7% were electives and 57.3% were emergency CSs [22]. These births are managed by consultant obstetrician gynaecologists, residents, house officers and midwives.

### Sample size calculation

Sample size for the quantitative study was calculated using the standard cross-sectional study sample size formula with a knowledge of CS among mothers found in another setting (13% of mothers knew their CS indications), at 95% confidence interval with a precision of 5% yielded a minimum sample size of 174 participants [6, 23].

### Data collection

Data collection consisted of qualitative and quantitative methods. It was done in phases.

### Inclusion and exclusion criteria

Postnatal women were eligible for the both the qualitative and quantitative study if they attended the post-natal clinic and had their first CS in the previous 6 weeks at the Korle-Bu Teaching Hospital. Women below 18 years were excluded. Those with very high blood pressures and severe anaemia were excluded because they needed urgent medical care. Health care professionals (including midwives and doctors) were selected based on their involvement with CS and having adequate level of work experience at the study site, i.e., at least 1 month of working at the Obstetrics Department.

### Phase one- Pilot study

Semi-structured explorative in-depth interviews based on the interview guides were held with postnatal mothers who had had previous CS (*N* = 30) and health care professionals (*N* = 8) in a private room at Korle-Bu Teaching Hospital. Each interview lasted 15 to 45 min. For the mothers, the interviews addressed questions about whether they were treated differently (by health care professionals, family and community members) due to the CS. Furthermore, mothers were asked if they had enough information about CS, if they would have wanted something more to be communicated to them (expectations), what SDM meant to them, and how they perceived the SDM process. Participation of the women (activation) was explored with questions on the willingness of a mother to be involved in decision-making [24]. Communication was explored with questions about time given to women to consider information and support provided in considering options by health care professionals [16].

For health care professionals, interviews addressed questions on how they thought women perceived a CS, how the SDM processes went and what factors may have affected SDM. During both interviews, there was space for women and health care professionals to discuss concepts that were not expected by researchers.

Following these initial interviews, the views of these stakeholders were considered and fed into the design of an interview guide for the focus group discussions and a questionnaire for the quantitative study, allowing for transdisciplinary research.

### Phase 2-Pretesting

The questionnaire and interview)guide were pretested among 10 mothers who presented for their 6 weeks postnatal review and 5 healthcare professionals at the Maternity Unit of the Department of Obstetrics and Gynaecology. The responses received from the pretests helped us to modify the final questionnaire to enhance its validity. Reliability of the quantitative questionnaire was calculated. A Cronbach’s alpha of 0.86 was obtained.

### Phase 3-Focus group discussions

Purposive sampling was used to recruit eligible participants into the qualitative study. We approached six mothers who had delivered by CS and had come for their 6 weeks postnatal review. We had a focused group discussion with them after they had given informed consent and had been attended to by the consulting doctors for the day. We subsequently approached health workers and grouped them into two groups of six each for focus group discussion.

**Fig. 2:**
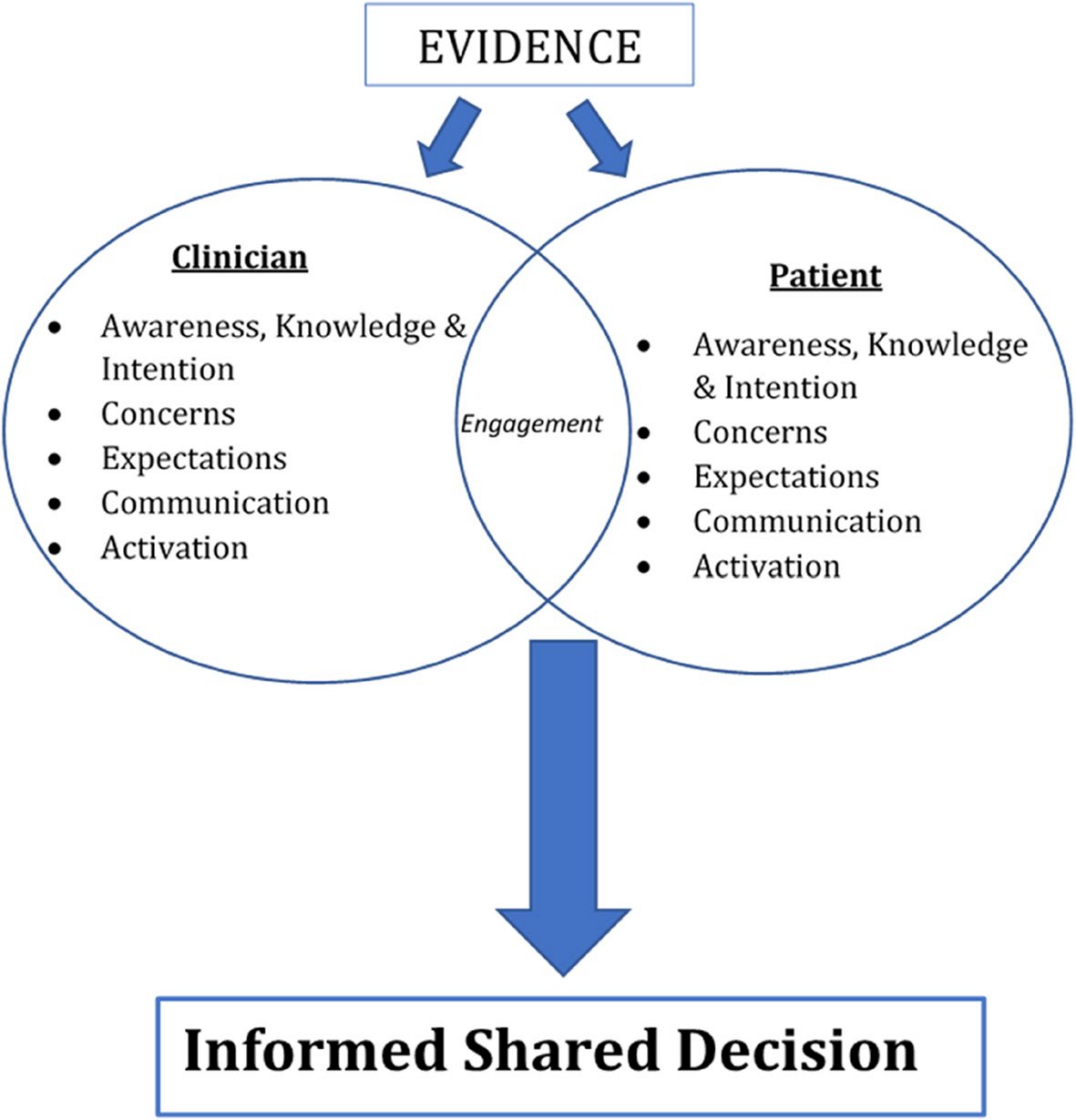
Adapted conceptual framework for informed SDM through engagement model by Moore et al. [16]

Focus group discussions for mothers and health care professionals were held in private rooms to allow for privacy and reported without directly attributing quotes to specific names. Focus group discussions aimed to explore prerequisites for SDM such as stakeholders, activation, communication, and information needed to make a decision (Fig. 2). Focus group discussions for the postpartum mothers were held in English and the two common local languages, Ga and Twi to enable them express themselves well in any of the languages they were comfortable to speak. Also, in addition to recording participants’ responses using an audio-recorder, a field note book was used to record additional information including non-verbal cues which would not be captured in the audio-recordings.

The first focus group discussion consisted of six mothers with ages ranging from 22 to 42 years, who all had living children except one who lost her child after CS. This group included one unemployed mother, one seamstress, one public worker and three traders. The second focus group discussion included six health care professionals comprised of one senior resident, two junior residents, and three house officers with a range of 5 months to 17 years professional experience with CSs. These different levels of doctors provided different perspectives from the doctors as they all interact with the patients before Caesarean section but play different roles in the decision-making process. The third focus group discussion with six health care professionals comprised of only house officers with 6 months to one year of professional experience with CSs. There were no refusals to participate in the focus group discussion and there were no drop-outs as well.

### Phase 4- Quantitative study

1. Interviews using questionnaires were held among mothers who were reporting for their 6 weeks postnatal review. One hundred and eighty questionnaires were completed by mothers (two were excluded because of age under eighteen years and one with incomplete responses) leaving 177 questionnaires for analysis. For the quantitative study, we selected five out of the eligible women who attended the clinic on each study day until we recruited a total of 180 women. Of all the patients who report for clinic on a particular day, the maternal health record booklets of women who had a previous CS were numbered from 1 to the last person and a computer- generated random sequence was used to select 5 out of the total number of available per day. Women who present on a particular day for postnatal care are attended to by a particular team of healthcare personnel. This sampling method of the women therefore, ensured that equal numbers were randomly selected each week day to avoid the situation where majority of the patients would have been selected from just a single team of healthcare workers.

The questionnaire for the quantitative study included sociodemographic characteristics, obstetric and gynaecological history, knowledge about CS, perceptions about CS, the actual Birth Decision Making Score and the willingness to be more involved in the decision making for CS. The actual Birth Decision Making Score was included to assess the satisfaction and involvement of mothers in the decision-making process of their CS [24]. The scale contains 6 questions with ‘true’ and ‘false’ as possible responses. Each response that is true is scored as ‘1’ and false is scored as ‘0’ and the sum total of all responses represented the level of satisfaction and involvement women perceived for their SDM [25]. The scores were dichotomized into ‘0–3’ (not satisfied with SDM) and ‘4–6’ (highly satisfied with SDM). Quantitative data was entered with Kobo Toolbox [26].

**Fig. 3:**
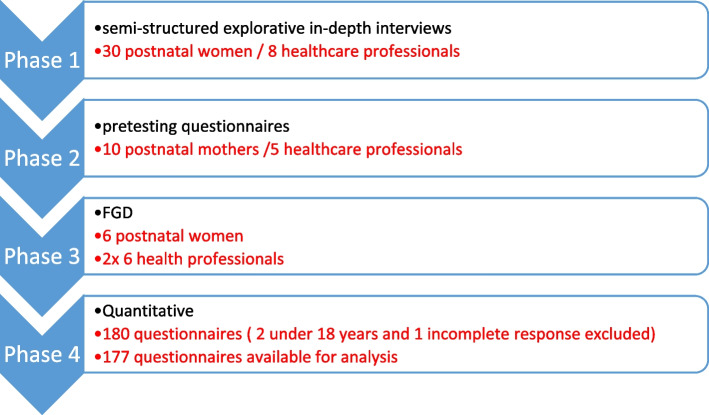
Flow chart showing the order of participant recruitment for various phases of the study

The flowchart showing the sequence of recruitments of participants into the study is shown in Fig. 3.

### Bias

Clearly defined eligibility criteria were decided before study started and was adhered to, to avoid selection bias. Information bias was minimized by having a week’s training for interviewers, back translating the questionnaires and piloting the questionnaires which also helped to maintain standardization of information.

### Analysis

First, interviews and focus group discussions were transcribed verbatim by interviewers. The nuances or meanings from the transcriptions were crosschecked with mothers and health care professionals where possible. Data from the interviews and focus group discussions were managed and analyzed using ATLAS.ti 8 (free trial version). Thematic framework analysis was used, based on research questions and conceptual framework, to analyze transcripts of participants, while deriving codes and categories. The qualitative results were analyzed and used in developing the quantitative tools by providing information and ideas that helped in the formulation of questions.

Secondly, descriptive analysis was performed using frequencies, percentages, mean and standard deviation for normally distributed data and median and interquartile range for data that was not normally distributed. Bivariate analysis was used to assess associations between independent variables and the dichotomized scores on the Birth Decision Making Score. This analysis included two-way tabulation for Pearson’s chi-square test or Fisher’s exact test when the former’s test for assumptions was not met. Multiple logistic regression was carried out to determine the factors associated with the desire for shared decision making and shared decision-making process scores. In all the statistical analyses, a confidence interval of 95% at a *p*-value less than 0.05 was considered statistically significant. STATA IC 15 was used for statistical analyses.

Following the analysis of the different data collected, we interpreted the entire qualitative and quantitative data. Results of both methods were outlined and checked for corroboration or contradiction.

## Results

### Characteristics of mothers who participated in the quantitative study

The sociodemographic characteristics of the mothers who participated in the quantitative study are shown in Table 1.

**Table 1:** Sociodemographic characteristics of mothers that responded to questionnaires

Variable	N (%)
	**Total = 177**
**Age**	
** Mean (S.D)**	30.6 (5.7)
**Age (in groups)**	
** 18 – 35**	133 (75)
** 36 – 45**	44 (25)
**Marital status**	
** Single**	7 (4)
** Co-habiting/engaged**	21 (12)
** Married**	149 (84)
**Religion**	
** Muslim**	20 (11)
** Christian**	156 (88)
**Level of education**	
** No formal education**	5 (3)
** Primary**	12 (7)
** Junior secondary school**	64 (36)
** Senior secondary school**	36 (20)
** Tertiary: vocational**	9 (5)
** Tertiary: professional**	17 (10)
** Tertiary: university bachelor**	29 (16)
** Tertiary: university master**	3 (2)
** Tertiary: Higher**	2 (1)
**Employment status**	
** Student**	3 (2)
** Unemployed**	9 (5)
** Salaried worker (private)**	21 (12)
** Salaried worker (public)**	23 (13)
** Artisan**	54 (31)
** Trader**	65 (37)
** Other**	2 (1)

### Knowledge of CS among mothers

Generally, mothers depicted a high level of knowledge of the medical indications for their CS, with 168 (95%) of respondents who knew the indication for their CS (Table 2). However, most mothers did not know about the existence of medications to relieve pain. In the interviews, most mothers were able to answer questions about indications for their CSs. However, in the focus group discussions, mothers expressed the desire to know even more about their CS indications. When asked about the indication for her CS, one woman said –

“…Yes! Abruptio Placentae – the placenta was torn”, but in the focus group discussion, she shared

“….I am sorry I may be diverting but I want to know what causes Abruptio Placentae since that was the indication for my CS and the loss of my baby?...”

**Table 2:** Knowledge of mothers that underwent primary CS

**Variable**		**N(%) Total = 177**
**Knowledge**		
** Knowledge about labour**	**No**	121 (68)
** Analgesia**	**Yes**	56 (32)
** Knowledge about Epidural**	**No**	158 (89)
** Analgesia**	**Yes**	19 (11)
** Knowledge about indication for CS**	**No**	9 (5)
	**Yes**	168 (95)
** Source of information about CS**	**Friends**	71 (40)
	**Books**	4 (2)
	**Doctor**	40 (23)
	**Family**	31 (18)
	**Internet**	21 (12)
	**Others**	4 (2)

Another woman said:

“…the reason was, the induction failed. And even with the induction it was because I was

having gestational diabetes…”

On the other hand, another mother was not quite satisfied with the information she got, she said.

*“….*actually, I’d have loved to know like, the complications it comes with. The bad, the negative sides of it. I’d have loved to know before….”

Another woman said:

“…. I wish they could have told me much more about the risks before making me do it”

During the focus group discussion women further explained that they would like to be better informed on all aspects about CS including their indications in simpler terms, measures to avoid it and related costs. They also stated that the best time to get this information would be during the preconception and antenatal period and it would be best shared by the doctors.

“…I would also want the healthcare provider to break down technical medical language, so I understand what is happening”

“… I would want to visit an obstetrician and ask all the necessary questions even before I become pregnant so that I can take precautions against developing the condition that led to the CS again.”

However, the health care professionals believed mothers did not know enough about their CS. This included indications, alternatives to CS if available, the procedure, possible complications, the recovery process and effects on future births. They attributed this to a low level of education among women, which affected their level of understanding of health information. Health care professionals further clarified in the focus group discussions that in their view, although most procedures were well explained to mothers, they found it difficult to re-iterate when later asked.

Forty percent of the mothers had their source of information on CS from friends compared to 23% who had their information from doctors (Table 2).

### Perception of CS among mothers

In general, CS was considered by women to be dangerous, ‘unnatural’ based on religious beliefs, and as a procedure that ‘takes away their strength’. The perceived diminished strength causes others in the community to perceive mothers as lazy, weak and fragile. One mother said:

“… some people will judge like they’re not women (i.e., the mothers) or they’re not strong enough to have babies the natural way.”

During the interviews, mothers expressed implicit concern and fear about CS and stated that it was not their preferred way of birth but, they underwent it because they thought it was the only option to save them and their baby’s lives. When asked, why CS was scary to them, one mother said:

“…I had heard there was the possibility of losing one’s life”.

Women also found CS to be unnatural according to their religion. One woman said -

“The best way to deliver is like the Hebrew women (vaginally), that’s what is in the Bible”.

The fear of mothers for CS and their perception of CS as being unnatural due to religious beliefs were also recognized by health care professionals during interviews. One house officer said:

“They [the women] say CS is not the ideal plan of God”

Health care professional**s** further explained that for these reasons, they face a lot of resistance from the mothers such as not agreeing to have CS or not showing up for antenatal visits. One senior resident explained:

“Women still are scared when they hear the word operation. So scared that some go and see pastors and the pastors would convince them they would deliver on their own (and these women wait) until complications set in.”

Over a quarter of mothers found CS to be scary. Despite the fear and the vast majority (95%, *n* = 169) of all mothers preferring vaginal birth to CS, 86% (*n* = 153) of mothers underwent CS because they thought it was their only option (Table 3).

**Table 3:** Perceptions of mothers that underwent primary CS

**Variable**	**N (%) Total = 177**
**Perceptions**	
** Preferred route of birth**	
** Vaginal birth**	169 (95)
** CS**	6 (3)
** Either one**	2 (2)
** Reason for agreeing to undergo a CS**	
** Previous negative birth experience with vaginal birth**	1 (1)
** I wanted a CS**	7(4)
** I was in pain**	11 (6)
** It was the only option**	153 (86)
** Other**	5 (3)
** Thoughts about CS as a mode of delivery**	
** It’s not good**	9 (5)
** It’s scary**	47 (27)
** It’s good**	97 (55)
** Other**	24 (14)
** Reasons why CS is offered to patients**	
** Safety of mother**	1 (1)
** Safety of child**	4 (2)
** Safety of both**	170 (96)
** No idea**	1 (1)
** How women who undergo CS are viewed by others**	
** Fragile**	9 (5)
** No idea**	20 (11)
** Normal**	22 (12)
** Weak**	93 (53)
** Other**	33 (19)

To minimize the fear and resistance of mothers, health care professionals proposed in the focus group discussions that religious leaders should be educated on the different means of childbirth and how CS does not make a woman less of a mother. In the interviews**,** some mothers reported that they were judged by people in the society as ‘not being women enough’ for not going through the ‘normal birth’. They further explained inn the focus group discussions that, because they will be judged, they hide the fact that they have undergone a surgery. During the health care professionals’ interviews, one resident explained further, saying -

“Such women are seen as weak and unhealthy especially if they are "rivals" in a polygamous marriage, the husband and other wife may often consider them to be weak”.

Questionnaire responses substantiated this finding as 53% (*n* = 93) and 5% (*n* = 9) of mothers reported that they were viewed in the society as being weak or fragile respectively, after undergoing a CS (Table 3).

### Factors contributing to SDM

During the interviews, 26 out of 30 mothers indicated that they were not aware of the concept of SDM. SDM was then conceptualized in context as having received adequate information about CS and taking part in and understanding the decision-making process. This was explained to the women before the focus group discussions. The focus group discussions therefore concentrated on elements of SDM such as stakeholders, women participation (activation), communication and information needed to make a decision. These elements served as themes for factors contributing to SDM in addition to time as an emerging theme.

### Effect of time on SDM

Insufficient time posed a challenge to SDM according to health care professionals and mothers. The lack of time adds pressure on all stakeholders to decide. During interviews, some mothers stated that it was difficult to have adequate communication with health care professionals because there was not enough time to consider options, think about the procedure, or its risks and benefits. One mother said:

“Because they were in a hurry I didn’t talk [ask]. If I talk (asked), they might tell me oh…”

One doctor also explained in the focus group discussion:

“…in emergency cases, not as much people [are] involved, it is not really shared [decision making].”

Moreover, one house officer explained that when there is enough time, a better SDM process is easily achieved:

“For elective cases, there is always enough time to engage them [mothers]. The pros and cons of the options are explained to her and she is given time to decide. She is made to understand that the decision ultimately lies with her, but she is free to consult anyone for guidance”.

The solution proposed by health care professionals to the time constraints was to better anticipate emergency cases. This can be achieved through proper antenatal monitoring of mothers and early referrals from other hospitals. Mothers were more likely to be satisfied and involved with SDM if they underwent an elective CS compared to emergency CS (*p* < 0.001). All mothers (100%, *n* = 48) that underwent elective CS were highly satisfied with their SDM process (Table 4).

**Table 4:** Factors associated with shared decision making for Caesarean section using the DDMS score

**Independent variables**	**DDMS score**		
	**0–3 N(%)**	**4–6 N(%)**	***p*-value**
**Age**			
18 – 35	25 (19)	108 (81)	0.093^*^
36 – 45	3 (7)	41 (93)	
**Marital status**			
Single	4 (57)	3 (43)	0.015^*^
Co-habiting/engaged	4 (19)	17 (81)	
Married	20 (13)	129 (87)	
**Religion**			
Muslim	24 (15)	132 (85)	1.000^*^
Christian	3 (15)	17 (85)	
**Highest level of education**			
< Tertiary	19 (16)	98 (84)	0.831^**^
≥ Tertiary	9 (15)	51 (85)	
**Antenatal visits**			
2–4	1 (9)	10 (91)	0.048^*^
5–9	19 (22)	66 (78)	
≥ 9	7 (9)	71 (91)	
**Type of CS**			
Elective	0	48 (100)	< 0.001^*^
Emergency	28 (22)	100 (78)	

^*^Fishers exact test

^**^Chi-square test

### Women participation (Activation)

The interviews revealed that some women were less willing than others to be involved in the decision-making process. One woman said:

“Anything the Doctor says, I will abide by it”

While, another mother was more inquisitive and said:

“I did not want to sign the consent form. They did not tell me what I was signing.”

However, during the focus group discussion, more women seemed more willing to enquire about their care and decision-making process when they were given the opportunity to. The difference in patient participation (activation) was explained during focus group discussion by 6 health care professionals as, educated women were perceived to be more interested in being involved in the decision-making process in comparison to less educated women. However, in the quantitative analysis, education of mothers was not found to be associated with SDM (Table 5). There was a significant association between number of antenatal visits and the desire to be more involved in SDM. The more antenatal care visits women had, the lesser the desire to be more involved in the SDM process. AOR = 0.091 95%CI (0.02–0.46) (Table 6).

**Table 5:** Multiple logistic regression for factors associated with a higher score on the Delivery Decision Making Scale (DDMS) for shared decision making for Caesarean section

Independent variables	Crude ODDs Ratio (95% C.I)	*p*-value	Adjusted ODDs Ratio (95% C.I)	*p*-value
**Age group**				
18 – 35	1		1	
36 – 45	0.32 (0.09–1.10)	0.071	2.33 (0.61–8.95)	0.218
**Marital status**				
Single	1		1	
Co-habiting/engaged	8.60 (1.79–41.31)	0.007	6.57 (0.89–48.11)	0.064
Married	1.52 (0.46–4.97)	0.491	4.91 (0.93–25.91)	0.061
**Religion**				
Muslim	1		1	
Christian	1.07 (0.29–3.94)	0.915*	1.01 (0.25–4.03)	0.989
**Educational level**				
< Tertiary	1		1	
≥ Tertiary	1.10 (0.46–2.60)	0.831**	0.83 (0.31–2.21)	0.710
**ANC visits**				
**< 5**	1		1	
**≥ 5**	0.53 (0.07–4.29)	0.999	0.66 (0.06–7.71)	0.738
**Type of CS**				
Elective	-	< 0.001*	-	0.997
Emergency			1

**Table 6:** Multiple Logistic regression for factors associated with desire to be more involved in shared decision making for Caesarean section

Independent variables	ODDs Ratio (95% C.I)	*p*-value	Adjusted ODDs Ratio (95% C.I)	*p*-value
Age				
18 – 35	1		1	
36—45	1.45 (0.72–2.93)	0.301	1.58 (0.70–3.59)	0.270
Marital status				
Single	1		1	
Co-habiting/engaged	0.80 (0.12–5.21)	.0.031	1.05 (0.15–7.43)	0.954
Married	0.16 (0.03–0.84)	0.001	0.13 (0.02–0.79)	0.027
Religion				
Muslim	1		1	
Christian	3.32 (0.93–11.8)	0.064	2.80 (0.69–11.28)	0.148
Highest level of Education				
< Tertiary	1			
≥ Tertiary	1.36 (0.70–2.64)	0.372	1.07 (0.49–2.33)	0.855
Antenatal visits				
< 5	1		1	
≥ 5	0.98 (0.02–0.47)	0.001	0.09 (0.02–0.46)	0.004
Type of CS				
Elective	0.93 (0.46–1.88)	0.847	1.28 (0.57–2.88)	0.545
Emergency			1	

### Stakeholders

When asked in the interviews about who should be involved in CS decision-making, mothers mentioned their husbands, a close relative and a familiar doctor. Health care professionals further explained in focus group discussion that mothers usually have a low self-esteem to make decisions on their own. Health care professionals also reported that most women depended on their husbands to make decisions, and even in dire emergency cases, some mothers would wait for their husband’s permission. Health care professionals suggested that a possible solution to this dependence on husbands, is to empower women, by educating girls and equipping women to provide for themselves financially. Health care professionals also stated that mothers should be educated on the possibilities of CS with a description of the procedure during antenatal care. One house officer said:

“If possible, husbands should also be involved in the antenatal stages.”

Quantitative analysis supports mothers’ preference for husbands to be involved in decision making. Eight out of ten mothers 81% (*n* = 154) thought that the decision to have a CS should include husbands.

## Discussion

In this mixed methods study within a tertiary facility in Ghana, most women who had recently had a CS knew the reason why this was done although little knowledge about post-CS pain relief was present. The perceptions about CS were often negative, with CS considered dangerous, ‘unnatural’, and a procedure that takes away their strength. This perceived diminished strength causes others in the community to perceive mothers as lazy, weak and fragile. Factors found to affect SDM were availability of time, patient participation (activation) and stakeholders such as religious leaders in the decision-making process. Little time for interaction between patients and doctors hampered the SDM process, for example during emergency situations. Women indicated a high willingness to engage in SDM, and wanted to involve their husbands.

Twenty seven percent of the women indicated that they were scared about CS because they feared losing their lives. This could reflect poor knowledge and professional public health education. It could also be due to the fact that Korle-Bu Teaching Hospital is a referral centre with a high-risk population and associated high institutional mortality rate. Sometimes very high-risk patients are referred to this tertiary facility. Unfortunately, some lose their lives after Caesarean section. This may not be linked to the surgery but to the conditions that they present with and the delay in getting professional help. This may send signals to ordinary people in the community that Caesarean section is a dangerous procedure.

A major source of information about CS seems to be from friends who could exaggerate the negative effects of the procedure. Our observation that the majority of women preferred a vaginal birth is similar to a study in Kumasi [6]. However, in contrast to this study, the majority of mothers in this study thought that CS was for the safety of both mother and child [6].

While the belief that vaginal birth is ‘normal birth’ and could contribute to the reduction of CSs with fewer requests based on non-medical reasons, this also contributes to negative perceptions and stigma about women who undergo CS, portraying them as ‘abnormal’ [14]. Similar to other studies from Ghana, mothers highlighted that they were perceived by others in the society as being lazy for undergoing CS and afterwards becoming weak and fragile [7, 8]. The role of religion further compounded these perceptions. We also observed similar gender dynamics as in another study on gender roles in CSs that found that women fear CS because they may be considered weak as observed by others – including husbands who in turn marry another wife to bear them more children [27], or other wives in the case of polygamous marriages.

The role of religion in decision making and possible delays in obstetric management and CS observed by health care professionals in this study when women may delay going to the hospital for CS after being convinced by pastors that they will deliver vaginally, was also found in Nigeria [27]. This contributes to the high number of women presenting to the hospital as emergency cases (72%), where there is less opportunity for information provision and shared decision making. Religious leaders need adequate education on the fact that both vaginal birth and CS are safe means of birth when indicated.

The majority of the women got information about CS mostly from their friends instead of doctors. This suggests that doctors should spend more quality time to explain the procedure and consequent effects and request feedback. This will also help erase the misconceptions that women have regarding CS and help alleviate the fears associated with CS. Appropriate scheduling of patients for antenatal clinic would give the doctors adequate time to interact with the women. The lack of time could be addressed by taking appropriate measures such as the use of decision making tools (written or audio), counselling women in groups and also making information about CS and the SDM process available online [28, 29].

Some mothers were more willing than others to be in involved in SDM. Health care professionals attributed this to their level of education. However, this could not be confirmed in the quantitative study.

SDM as a concept may be unfamiliar with a number of women in lower middle income countries and there should be continuous education of general public on the benefits to be derived from it. While attempts are made to improve on the concept of SDM in this context, it is always important to emphasize the broader overarching patient- centered communication which ensures that patients understand steps taken at all levels in the care spectrum. The number of antenatal visits may serve as a proxy to assess willingness of mothers to be involved in their healthcare decision. In this study, we found that the more antenatal care visits, the more satisfied and involved mothers were in the SDM of their CS and consequently have a lesser desire to be more involved in the SDM process. A conscious effort should be made by health care providers to start engagement of antenatal care attendants early and to maintain this throughout the pregnancy, birth as well as the postnatal period.

Most women wanted their husbands to be involved in the SDM process and this should serve as a guide to Health care professionals when they go through the process with women. That is, if possible, and with the women’s permission, husbands be involved in the SDM process. Health care professionals emphasized how dependent mothers were on their husbands to accept to undergo CS. This dependency may be because the husbands are responsible for bearing financial costs of the CS as was found in another study, that there is pressure on mothers from their husbands to have vaginal births [27]. To address this issue of dependence, health care professionals suggested that the empowerment of women could play an important role in the SDM process as mothers can take more responsibility for their health [30]. Empowerment should include health care professionals providing mothers with enough information to take decisions on their own and recognizing that mothers have ultimate control over their own health [30]. Mothers were mostly satisfied with the decision-making process and they preferred a joint decision including at least one familiar doctor. They expressed essential topics to be covered in adequate counseling, information provision and decision making, including information about the procedure in general, CS indications, how CS will be carried out, post procedure complications, recovery, as well as effects on future births. This is important because mothers having more information may significantly increase their level of satisfaction with their healthcare as seen in a study in Karachi [31–33].

Although, vulnerable people, such as less educated women, are more likely to benefit from engaging in SDM, they are less likely to want to partake in it as was also explained by health care professionals in this study [34]. Power differentials between health care professionals and these women may contribute towards them not wanting to take part in SDM. This behaviour of indifference is said to be modifiable [34] and was observed in the focus group discussions where mothers seemed to become more confident in their ability to partake in decision making. Health care professionals will need continuous professional education on how to engage vulnerable groups in a way that will enable open participation in decision making.

In this study, we found that there was a relationship between the type of CS (emergency vs elective) mothers underwent and their perceived satisfaction and involvement with the SDM. Similar to other studies, time constraint was reported to be a barrier to SDM by both mothers and Health care professionals [34]. However, other studies found no evidence that it takes significantly more time to engage in SDM compared to the normal care provided [34–37]. Therefore, an improvement in the decision-making process may not take as much time as perceived by mothers and health care professionals. Although, this study found that the lack of time did not allow mothers to ask doctors enough questions about their care, the reluctance to ask questions may also be due to fear of doctors’ negative reaction or being seen as difficult [34].

This study took a transdisciplinary research approach in which the first action in this research was to explore this specific context. This was done by collaborating with local researchers, local health care professionals and mothers. The main aim of the focus group discussion with mothers was to achieve knowledge co-creation amongst them while understanding their desires on the decision-making processes. A step-by-step participatory approach was taken with the mothers, by analyzing the decision-making process of a typical mother in Korle- Bu Teaching Hospital provided to them in the form of a story (formulated through reports of experiences during interviews). The focus group discussion with the mothers eventually turned out to be a learning process for them. This discussion provided the women with ‘a space’ to ask questions that they felt uneasy asking during doctor visits and educate themselves on what they did not know. It also made them more inquisitive about their health, which is needed for SDM. The interviews and focus group discussions encouraged mothers to ask more questions about their care from their health care professionals. The questions asked in the discussions like “what choices do I have?” “What are the risks and benefits?” have been found to improve the engagement between Health care professionals and patients (Patient mediated interventions) [32]. These key issues should be discussed with the women by the health care professionals to enhance a positive pregnancy experience. This communication about shared decision making needs to be done with due consideration to the cultural context within which these women live to improve upon women’s acceptance [38, 39].

### Strength and limitations

The study involved transdisciplinary research and a mixed methods design on SDM during CS which involved relevant stakeholders, which, to the best of our knowledge, is the first such study in the lower middle income setting. In this study, knowledge was generated and integrated among stakeholders during the research process as they learnt from each other’s experiences. Health care professionals were also made more aware of how women perceived their CS and their desires for SDM which could possibly be used to tailor future decision-making processes. The transdisciplinary research method encouraged health care professionals to analyze their clinical practices, identify things that may need improvement in the CS decision making process and understand the need for change.

Limitations included the fact that we were unable to cross-check the indications provided by mothers for their CS as the ones given by their doctors in order to identify if they truly remembered their medical indications. Another limitation was the language barrier which did not allow for having more than one focus group discussion with mothers because of the time needed to translate and transcribe.

From a clinical perspective, an issue that was not prominently mentioned in any interview or focus group was that of the risks during subsequent pregnancies, which are significantly raised in mothers with a CS scar.

## Conclusion

Mothers showed high levels of knowledge about the indications for their CS, but important misconceptions existed about CS among Ghanaian mothers and community members. There was a lack of awareness about the concept of shared decision making among mothers. However, during conversations more women were eager to obtain information about their CS. Factors that contributed to SDM were the willingness of mothers to be involved in decision making, husbands’ participation and sufficient time to make decisions. Religious leaders are also key stakeholders in SDM. Women who had more than four antenatal visits had a reduced need for SDM. Involvement of pregnant women and their husbands, if possible, in the decision-making process will enhance a positive pregnancy experience. There is the need to explore the use of written and audiovisual decision-making tools, group counselling on SDM and online information systems to increase the time for engaging women in the SDM process.

## Supplementary Information

**Additional file 1.** Focus group discussion guide.

## Data Availability

The hard copies and electronic data set are available from the corresponding author on reasonable request.

## Abbreviations

CS: Caesarean section
SDM: Shared Decision Making
WHO: World Health Organization
