# Supplementary material for: Exploring the shared decision making process of caesarean sections at a teaching hospital in Ghana: a mixed methods study
Source: BMC Pregnancy Childbirth. 2023 Jun 8;23:426. doi: 10.1186/s12884-023-05739-7 (PMC10249274; doi:10.1186/s12884-023-05739-7)
Supplement: Supplementary file 1 — Additional file 1. Focus group discussion guide. [file 12884_2023_5739_MOESM1_ESM.docx]

**Additional file 1: Focus group discussion guide**

Bold and Non-italics: Instructions to the facilitator

| **Purpose** | **To explore the needs of mothers to make informed decisions of CSs as a pre-requisite for Shared Decision Making (SDM) in caesarean sections (CSs).**  The sub-questions of this FGD are:   - What do mothers think about SDM in CS? (brief recap) - Is it desired/needed by mothers to be involved in SDM of CS? - What information given to mothers is most useful? - What questions would you like answered during your caesarean section journey? – can I tie my stomach? - What other information would mothers find useful to improve their experience? Why? - When is the best time to get this information? - What format would mothers like information provided in? | |
| --- | --- | --- |
| **Target population** | Mothers that had their first CS in their most recent delivery | |
| **Interviewers** | Onisarotu Nana Aisha, MD (researcher at VU, Amsterdam) and Anafo Godwin, MD (research assistant at KBTH). ONA was female, and AG was male. ONA’s MSc training at VU included abundant training in qualitative research, and has previously carried out interview-based studies in maternal health. AG was a medical doctor with hands on experience in the decision making process of CS. | |
| **Materials** | 5 flipcharts, 54 post-its, tape, 6 pens, 2 markers (2 different colors), 10 red stickers, 4 copies of all transcripts, room with long tables. | |
| **Room preparation** | • All facilitators arrive early to set up the room. This allows time to deal with unexpected occurrences and set up materials (prepare flip charts) and refreshments.  • Chair arrangements to accommodate 6 participants, 1 facilitator and 1 assistant (8chairs). • Post plenty of signs so participants can find their way to the space to help participants feel welcome when they arrive.   - Give participants name tags. | |
| **Duration** | **Content** | **Remarks/rationale** |
| **3 minutes** | **Opening/ Introduction of the topic**  Facilitator:  *Good morning everyone, I’d like to welcome you all to this focus group discussion.*  *Thank you for taking your time to be here with us today. We will be here for about thirty minutes give or take.*  *The reason we are here today is to discuss about the decision-making process of CS. It’s challenges and possible solutions.*  *My name is {name}. I will be leading the focus group discussion and my colleague here {name} will take notes and help with some technical issues.* | To welcome participants, inform them on practical issues, confidentiality and the purpose of the discussion.  **Goal of the section**: to introduce the interviewers, and the topic of FGD |
| **2 minutes** | ***Practical issues***  *To allow our conversation to flow freely, I’d like to go over some practical issues*   1. *On the table are some post its. Please use one to indicate your name and position and place it where it is visible to the rest of us.* 2. *There are no obligations during this discussion, but we will really appreciate your active participation.* 3. *There are no right or wrong answers, so feel free to speak your mind.* 4. *This is a confidential discussion and material gathered will only be shared among the research team members.* 5. *Please enjoy the refreshments on the table as the discussion goes on* 6. *We shall have a break midway if need be.*   *Are there any questions?* | To inform participants about practical issues regarding the FGD |
| **7 minutes** | *So, first let’s get to know each other.*  *Can we introduce ourselves, our jobs, say our obstetric backgrounds, how we would have preferred to deliver and how we delivered our last babies?*  *For example;*  *My name is {name}, I am a {occupation}, I have been pregnant {_} times, and I have {_} children. I would prefer to deliver via {_} and my last baby was delivered via {CS}.*  (**round of presentations**) | To get acquainted with each other and break the ice. |
| **10 minutes** | *Like I said earlier, SDM is going to be the major theme of our discussions today.*  *Please, can you tell me what comes to mind when we hear shared decision making in CS.*  *Let’s take some time to write on the post-its provided (3 per person) or just think about it and we will discuss after.*  **(present flipchart of pregnant Madam Mary and a HEALTH CARE PROFESSIONALS in the middle)**  Possible answer: When I hear shared decision making, I think about {mothers and doctors coming together to make decisions about the mother’s care.  *Would anyone like to share what they wrote?*  **(place post-its around the picture on the flipchart)**  *Who wrote something similar?*  *Who wrote something that they feel is associated to the one discussed?*  *Who wrote something different?*  *What do you think about SDM? Is it important? Is it needed? Is it desired?*  **(build a discussion around what has been found and put the flipchart through a popcorn method)**  (Probes: *Why do you think it should be only doctors? Why do you think it is also important to involve mothers?)*  ***(conclude session)***  *Ok, so now we have said what comes to mind when we think about SDM.*  *We can see that some of us mentioned communication or agreement between husband and wife and others said between Doctors and mothers...* | To help participants to get into the topic and reflect on how the subject of the FGD, SDM, is specific to each context. |
| **15 minutes** | **Solution generation**  *This shows that we all think of SDM is a similar/different way.*  *{name} mentioned that it is important to involve mothers because….*  *While keeping these ideas of SDM in mind, let’s move on to the next exercise.*  *For this section I am going to present you with a story and I would like if we could all share our opinions and actively participate.*  *I would ask you some questions at different points in the story and please remember that there is no wrong answer and respond freely.*  **(write the components of the different questions on the flipchart)**  *Is everyone ready to continue?*  *Madam Mary is 27years old. She lives in Kasoa. She is 36 weeks pregnant with her second baby. Her first baby was delivered via vaginal delivery without problems, so she is expecting to deliver the same way again. She came for her regular antenatal visit at the clinic but was told that she would have to be taken to the labor ward to deliver as soon as possible.*  *How do you think the doctor should explain the situation to her?*  Possible responses:   - The doctor should tell her why she must deliver now.   *Madam Mary has eclampsia (her blood pressure is very high)/ placenta abruptio which is why she has to deliver now. Madam Mary has been transported to the labor ward. While in the labor ward, the doctors tried to induce her labor with the help of the nurses, but the baby is not coming. She is not having adequate contractions.*  *What do you think happens next?*  Possible responses:   - They will continue to wait   *Madam Mary is told that her baby’s heart is not beating well, and her baby would die if she does not perform a CS.*  *How do you think Madam Mary is feeling? What is she thinking about? What questions would she like to ask?*  *Madam Mary is told about the risks and benefits of caesarean sections to her and her baby which are….*  *What do you think happens next?*  *Madam Mary is brought the consent form to sign by the nurse.*  *What does madam Mary do? …………… Does she sign the consent form? Sign the consent form only after she reads it? or asks the nurse to read it to her?*  *How do you think madam Mary feels about signing the consent form? Do you think Madam Mary wants more information?*  *Alright. So, madam Mary has been operated and is doing well with her baby.*  **(start from one post-it and place that on the paper)**  *In general, during this process, is there any other information that you find very useful to know? Something from your personal experience you would like to discuss?*  **(put all information and questions on the flipchart)**  *I would like you to try to think of these processes and your own personal experience while you fill in these papers.*  **(distribute paper for clinical experience process)**  *Please, indicate how you felt at these stages and reflect on them. Is there any information that could improve your experience? Any questions that you would like answered?* | To allow participants highlight different information that is useful for them at different points of their delivery experience. In addition, questions that they would like to have answered during their CS journey    **Goal to the section**: Most useful information for mothers during their CS journey |
| **5 minutes** | Break |  |
| **7 minutes** | **Solution Implementation**  *Ok, so we can see that we have *** groups of similar information we would like to know and questions we would like to be answered.*    *Let’s start from this first one, When do you think is the best time to get this information? During ANC visits?*  *In what format/way would you like to get this information? Media? From the doctors? Nurses?* | To explore mothers’ proposals for possible ways to implement solutions to improve clinical experience and knowledge.  This is done in pairs so that incase the solutions they provide are very different, participants can discuss together to understand each other’s perspectives of why they think the solutions are important. |
| **3 minutes** | **Feedback**  *So, now we are concluding the FGD, and we would like you to give some feedback about your experience in this FGD.*  *What did you think about the FGD?*  *Also, are there other areas that we did not discuss that you feel are also important?  Last, I would like you to name one thing you learned in this FGD.* | To ask them if they have learnt something to find out if there has been any knowledge integration or co-production.  Participants might have ideas about other areas that they feel are also important in SDM. |
| **1 minute** | **Closing**  *Thank you for your active participation.*  *Before you leave, we have a small gift for you, to thank you for your participation.*  *We are very happy about how the FGD went and we hope you enjoyed it too.* |  |

**53minutes (will be shorter)**

**Mothers will be asked to fill this in. Their responses will instigate some conversations of their personal experiences.**

**Your caesarean section delivery and shared decision making**

This page is intended to help you think about how you feel at different stages in your journey through the hospital. Please, tick the box next to the face which best represents your feelings at each stage

**Indication given for CS**

**Information given on CS**

**Consent form**

**ANC clinic**


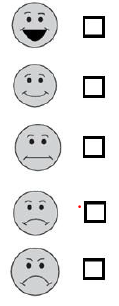

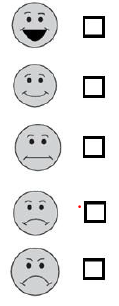

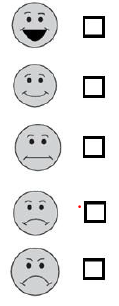

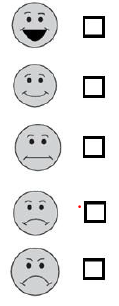


**How can we make it better?**
